# Pan-cancer analysis identifies RNF43 as a prognostic, therapeutic and immunological biomarker

**DOI:** 10.1186/s40001-023-01383-1

**Published:** 2023-10-17

**Authors:** Yingting Xu, Zhengjun Lin, Yuqiao Ji, Chen Zhang, Xianzhe Tang, Chuan Li, Tang Liu

**Affiliations:** 1 Department of Orthopedics, The Second Xiangya Hospital, Central South University, 139# Middle Renmin Road, Changsha, 410013 Hunan People’s Republic of China; 2 Department of Orthopaedic, 920Th Hospital of Joint Logistics Support Force of Chinese People’s Liberation Army, 212 Daguan Road, Xishan District, Kunming, Yunnan China; 3 Department of Orthopedics, Chenzhou No.1, People’s Hospital, Chenzhou, 423000 Hunan China; 4 Department of The Emergency, The Fourth People’s Hospital of Zigong, Zigong, 643000 Sichuan China

**Keywords:** RNF43, Pan-cancer, Immunotherapeutic efficacy, Prognostic biomarker, Immune microenvironment, Genetic alternations, Drug sensitivity

## Abstract

**Background:**

RING finger protein 43 (RNF43), an E3 ubiquitin ligase, is a homologous gene mutated in several cancers. However, the pan-cancer panoramic picture of RNF43 and its predictive value for tumor immune phenotypes and immunotherapeutic efficacy are still largely unclear. Our study aims to clarify the functions of RNF43 in predicting the prognosis, immune signature, and immunotherapeutic efficacy in pan-cancer.

**Methods:**

By using RNA-seq, mutation, and clinical data from the TCGA database, the expression levels and prognostic significance of RNF43 in pan-cancer were analyzed. The genetic alteration characteristics of RNF43 were displayed by the cBioPortal database. Gene Set Enrichment Analysis (GSEA) was performed to investigate the potential biological functions and signaling pathways modulated by RNF43 in cancers. The relationship of RNF43 expression with immune cell infiltration, and immune modulators expression was interpreted by the ESTIMATE algorithm, CIBERSORT algorithm, and TISIDB database. The correlations between RNF43, microsatellite instability (MSI), and tumor mutation burden (TMB) were also investigated. Furthermore, the predictive value of RNF43 for immunotherapeutic efficacy and drug sensitivity was further illustrated. Besides, immunohistochemistry (IHC) was employed to validate the expression of the RNF43 in different cancer types by our clinical cohorts, including patients with lung cancer, sarcoma, breast cancer, and kidney renal clear cell carcinoma.

**Results:**

The results demonstrated that RNF43 was abnormally expressed in multiple cancers, and RNF43 is a critical prognosis-related factor in several cancers. RNF43 was frequently mutated in several cancers with a high frequency of 4%, and truncating mutation was the most frequent RNF43 mutation type. RNF43 expression was linked to the abundance of several immune cell types, including CD8+ T cells, B cells, and macrophages within the tumor immune microenvironment. Furthermore, RNF43 expression was significantly correlated with the efficacy of anti-PD-1/PD-L1 treatment, and it could predict the sensitivity of various anti-cancer drugs. Finally, IHC explored and validated the different expression levels of RNF43 in different cancers by our clinical samples.

**Conclusion:**

Our results first present the expression pattern and the mutation signature of RNF43, highlighting that RNF43 is an important prognostic biomarker in pan-cancer. Furthermore, RNF43 seems to be a critical modulator in the tumor immune microenvironment and can function as a promising biomarker for predicting the immunotherapeutic efficacy of anti-PD-1/PD-L1 treatment, and drug sensitivity in cancer treatment.

**Supplementary Information:**

The online version contains supplementary material available at 10.1186/s40001-023-01383-1.

## Introduction

Cancer immunotherapy aims to activate the anti-tumor immune response to kill cancer cells and has greatly changed the paradigm of cancer treatment [1]. Nowadays, immune checkpoint inhibitors and chimeric antigen receptor T cell therapies have been commonly applied in clinical management [1]. However, the main obstacle to successful cancer immunotherapy is that cancer cells can develop the capability to evade immune system attacks, and the majority of cancer patients have innated or acquired immunotherapeutic resistance [2, 3]. Emerging evidence has identified that the infiltration of different immune cell types within the tumor immune microenvironment seems to broadly overlap with developing resistance to immunotherapies [4]. Furthermore, the mutations generated from genomic instability in cancer may induce different neoantigens and affect the immune cell infiltration in the tumor immune microenvironment, thus leading to cancer immunoediting and immunotherapy resistance [5]. Therefore, exploring novel biomarkers that are critically involved in regulating cancer immunogenicity and immune microenvironment can pave the way to understand the development of resistance to immunotherapies, and develop novel strategies to overcome such resistance.

Previous research has revealed that the dysregulation of the Wnt signaling pathway is fundamentally involved in tumorigenesis and cancer progression, and ubiquitination is a key regulator of the Wnt signaling pathways in physiological and pathological processes [6]. As an E3 ubiquitin ligase, RNF43 (E3 ubiquitin-protein ligase RNF43 or RING-type E3 ubiquitin transferase RNF43), which can be stimulated by the activation of the Wnt pathway, can ubiquitinate Wnt receptor complex components frizzled receptors, thereby suppressing the Wnt signaling pathway in turn [7]. The tumor suppressive roles of RNF43 in different cancer types have been identified in multiple studies. For instance, it has been found that RNF43 can attenuate the stemness of gastric cancer stem-like cells by inhibiting the Wnt/β-catenin pathway and impairing the chemoresistance in vitro [8]. Moreover, RNF43 is recurrently mutated in several cancer types, and RNF43 mutations can promote the initiation and development of human malignancies, such as gastrointestinal cancers, hepatocellular carcinoma, and pancreatic adenocarcinoma [9–11]. RNF43 loss-of-function mutations are often detected in MSI-type colorectal cancer, and PORCN inhibitor, which can inhibit ligand-mediated activation of the Wnt/β-catenin cascade, can effectively inhibit the progression of RNF43-mutant cell-derived PDX colorectal cancer in vivo [12]. By constructing a genetically engineered pancreatic ductal adenocarcinoma mouse model via conditional expression of oncogenic Kras and deletion of the catalytic domain of RNF43, loss of RNF43 promotes pancreatic ductal adenocarcinoma tumorigenesis, and contributes to decreased survival time of mice [13]. Notably, several clinical trials have confirmed the efficacy of RNF43-based immune therapies in colorectal cancer treatment to date [14, 15]. These findings present that RNF43 may be critically involved in cancer progression and tumor immune microenvironment remodeling. However, there is a lack of comprehensive pan-cancer analysis of the clinical value and genetic mutations of RNF43, and the exact roles of RNF43 in modulating cancer progression and tumor immune microenvironment are still largely unknown.

In the present study, we performed systemic research on the promising roles of RNF43 in predicting the prognosis and immune phenotypes in human cancers. We explored the clinicopathological significance and genetic mutation characteristics of RNF43, and the crosstalk between RNF43 expression and the abundance of immune cells and immune-related molecules in the tumor immune microenvironment. Besides, we further elucidated the predictive value of RNF43 for the immunotherapy efficacy and drug sensitivity in pan-cancer, thus highlighting the promising role of RNF43 as a clinical biomarker and a novel therapeutic target for cancer patients in clinic settings.

## Materials and methods

### Data collection

RNF43 expression data in pan-cancer was extracted from TCGA (The Cancer Genome Atlas), project name: TCGA-pan-cancer, and expression data in normal human tissues was obtained from the Genotype-Tissue Expression (GTEx) on UCSC Xena (https://xena.ucsc.edu/). The expression pattern of RNF43 in 24 different cancer cell lines from the CCLE database (https://portals.broadinstitute.org/ccle/about) was also investigated [16]. Differential expression between diverse cancer types and their corresponding normal samples were evaluated by Student T-test and visualized by R package “ggplot2”.

### Genomic alterations of RNF43 in pan-cancer

The genomic alteration characteristics of RNF43 in the TCGA pan-cancer dataset were on the cBioPortal database (https://www.cbioportal.org) [17]. The genetic alteration rate, mutation types, and mutated site information of RNF43 in pan-cancer were investigated. Genetic mutation co-occurrence analysis was also conducted.

### Prognostic analysis of RNF43 in pan-cancer

Cox proportional hazard models and Kaplan–Meier plotter analysis were conducted to evaluate the clinical value of RNF43 in predicting survival outcomes including overall survival (OS), disease-free survival (DFS), disease-specific survival, (DSS), and progression-free survival (PFS) in pan-cancer. The results were shown by forest plots and Kaplan–Meier curves.

### GSEA analysis of RNF43 in pan-cancer

To explore the underlying biological functions and signaling pathways of RNF43 in pan-cancer, GSEA was subsequently performed. Kyoto Encyclopedia of Genes and Genomes (KEGG) and Gene Ontology (GO) gene database were extracted from the GSEA website (https://www.gsea-msigdb.org/gsea/downloads.jsp). The analysis process was conducted by utilizing the R packages “limma”, “org.Hs.eg.db”, “enrichplot” and “clusterProfiler” with the following parameters: nPerm = 100, and p-value-Cutoff = 1.

### Correlation of RNF43 expression with the tumor immune microenvironment in pan-cancer

The Estimation of Stromal and Immune Cells (ESTIMATE) algorithm was performed to calculate the immune and stromal scores of each cancer sample by R package “estimate”. By using CIBERSORT, we investigated the correlation of RNF43 expression with the abundance of diverse immune infiltrating cell types in different cancers. The interaction between RNF43 expression and immune system-related modulators, such as chemokine, chemokine receptor, tumor-infiltrating lymphocytes (TIL), major histocompatibility complex (MHC), immune stimulator, and immune inhibitor in different cancers was also assessed in TISIDB online database (http://cis.hku.hk/TISIDB/index.php).

### Correlation of RNF43 expression with MSI and TMB in pan-cancer

The MSI and TMB data were acquired from TCGA pan-cancer mutation data. TMB is defined as the corrected number of mutant bases per million bases. MSI is a molecular phenotype caused by deficient DNA mismatch repair activity. Spearman’s method was carried out to evaluate the correlations between the expression level of RNF43 and MSI/TMB. Radar plots by the R-package “fmsb” were utilized to visualize the results.

### Correlation of RNF43 expression with the immunotherapeutic efficacy

To evaluate the correlation between RNF43 expression and the efficacy of immunotherapy, we enrolled three datasets comprising samples who were treated with anti-PD-1/PD-L1 therapy, including GSE78220 (melanoma), GSE67501 (renal cell carcinoma), and IMvigor210 (metastatic urothelial cancer) from GEO (https://www.ncbi.nlm.nih.gov/geo/). The investigation procedure was conducted and the results were visualized by utilizing R-package “ggpubr” and “ggplot2”.

### Correlation of RNF43 expression with drug sensitivity

To elucidate the correlation between RNF43 and drug sensitivity in pan-cancer, NCI-60 compound activity data with RNA-seq expression data were downloaded from the CellMiner™onlinde database (https://discover.nci.nih.gov/cellminer/home.do). The correlation between RNF43 and the sensitivity of drugs approved by the FDA was analyzed by utilizing R packages “impute”, “limma”, “ggplot2”, and “ggpubr”.

### Clinical samples and immunohistochemistry

We obtained clinical samples for our study through a protocol approved by the Second Xiangya Hospital. Following the Declaration of Helsinki, all patients gave informed consent. We stained both clinical tumor tissue samples and commercially available tumor tissue chips for RNF43 using an IHC staining method, which was implemented with RNF43 antibody (1:50; Proteintech, China) according to the manufacturer's protocols. The tumor tissue sections underwent deparaffinization and subsequent rehydration. The antigen was immersed in pH = 6.0 citrate buffer for 15 min at 95 ℃. Subsequently, the activity of endogenous peroxidase was blocked by incubating the sections with 0.3% hydrogen peroxide for 15 min at room temperature. Eventually, the antigen was retrieved. Then, we blocked the sections with PBS rinsing and 5% normal goat serum at room temperature for 30 min before applying the primary anti-RNF43 antibody overnight at 4 ℃. We counted the proportion of negative (–), weakly positive ( +), moderately positive (+ +), or strongly positive (+ + +) staining cells, as well as the intensity of cell staining in five randomly selected fields.

## Results

### RNF43 expression analysis in pan-cancer

**Fig. 1:**
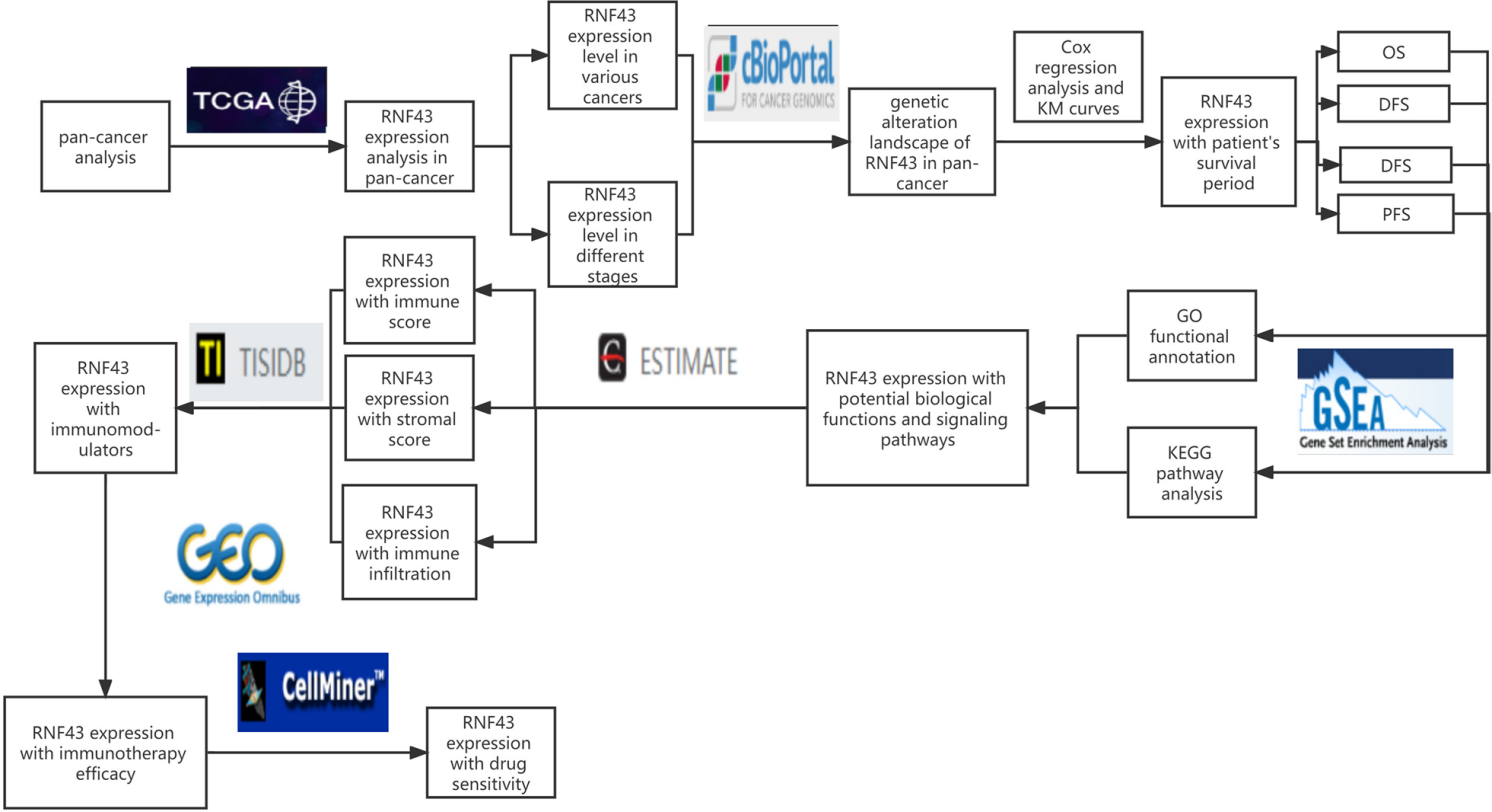
The workflow of the present research

**Fig. 2:**
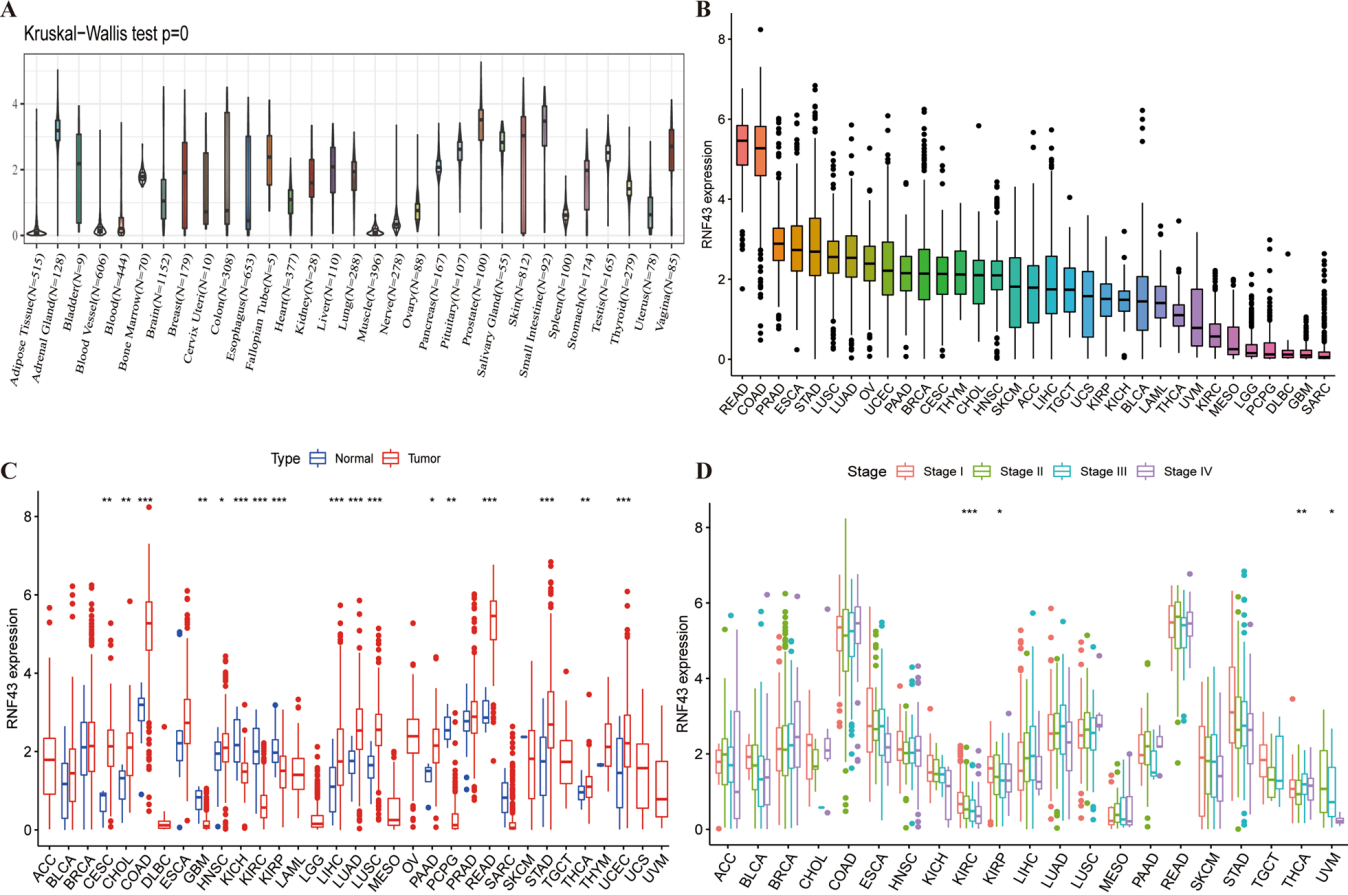
The expression pattern of RNF43. **A** RNF43 mRNA expression in 31 normal tissues in the GTEx database. **B** RNF43 mRNA expression in cancers in TCGA database. **C** Differential RNF43 mRNA expression between cancer samples and compared normal tissue samples in the TCGA database. **D** Differential RNF43 mRNA expression in different clinical stages in TCGA database. *p < 0.05, **p < 0.01, and ***p < 0.001

The workflow of our investigation was shown in Fig. 1. Firstly, we investigated RNF43 expression in normal tissues in the GTEx database and cancer cell lines in the CCLE database. The RNF43 was differently expressed in diverse normal tissues and cancer cell lines. RNF43 was highly expressed in the large intestine and stomach samples, while it was expressed at low levels in several normal tissues, especially adipose tissue and muscle tissues (Fig. 2A). To explore the expression pattern of RNF43 in different cancers, we studied RNF43 expression in various cancers from the TCGA pan-cancer dataset. RNF43 was highly expressed in rectum adenocarcinoma (READ), and colon adenocarcinoma (COAD), whereas was lowly expressed in lymphoid neoplasm diffuse large B-cell lymphoma (DLBC), glioblastoma (GBM) and sarcoma (SARC) (Fig. 2B). Next, the differential RNF43 expression between cancer samples and their compared normal samples in the TCGA pan-cancer dataset was further analyzed. A significant expression difference of RNF43 was found in 33 types of cancer excluding those without normal tissue data. RNF43 expression was overexpressed in cervical squamous cell carcinoma and endocervical adenocarcinoma (CESC), cholangiocarcinoma (CHOL), colon adenocarcinoma (COAD), head and neck squamous cell carcinoma (HNSC), liver hepatocellular carcinoma (LIHC), lung adenocarcinoma (LUAD), lung squamous cell carcinoma (LUSC), pancreatic adenocarcinoma (PAAD), rectum adenocarcinoma (READ), stomach adenocarcinoma (STAD), thyroid carcinoma (THCA), and uterine corpus endometrial carcinoma (UCEC) compared to the normal tissues (Fig. 2C). On the contrary, RNF43 was decreased in glioblastoma multiforme (GBM), kidney chromophobe (KICH), kidney renal clear cell carcinoma (KIRC), kidney renal papillary cell carcinoma (KIRP), and pheochromocytoma and paraganglioma (PCPG) compared to the normal tissues (Fig. 2C). Furthermore, our results revealed that RNF43 was significantly associated with the clinical stage in 4 cancer types, in which RNF43 expression levels were lower in later clinical stages, including KIRC, KIRP, THCA, and uveal melanoma (UVM) (Fig. 2D). These findings revealed the abnormal RNF43 expression pattern in pan-cancer and the clinical significance of RNF43 in predicting tumor stage progression.

### The genetic alteration landscape of RNF43 in pan-cancer

**Fig. 3:**
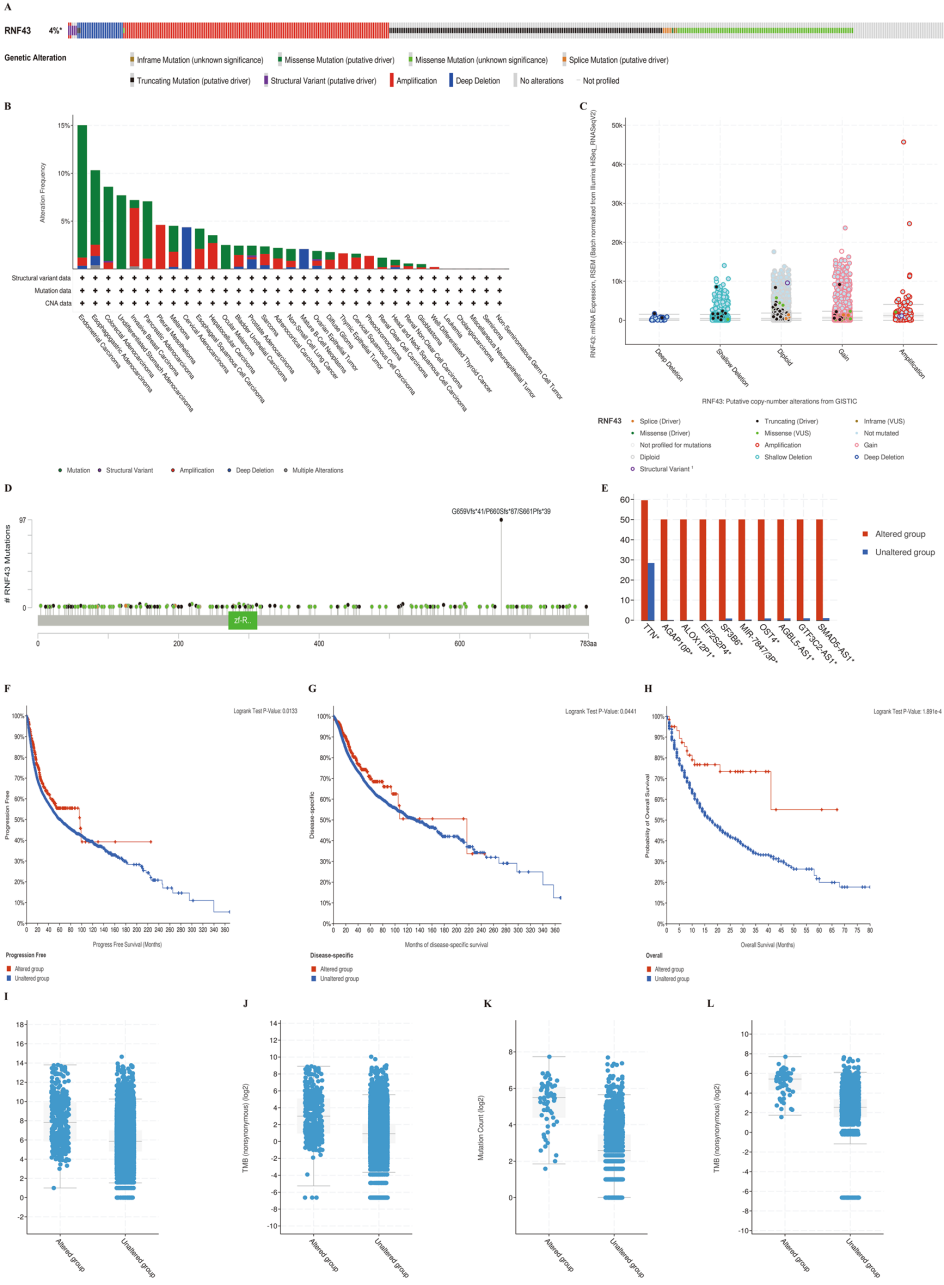
The genetic alterations of RNF43. **A** Summary of RNF43 structural variant, mutations, and copy-number alterations. **B** Summary of RNF43 alterations in TCGA pan-cancer cohort. **C** The putative copy-number alterations of RNF43 in pan-cancer. **D** The mutation number, type, and site of the RNF43 genetic alterations. **E** The cooccurrence of gene alteration in RNF43 altered group and unaltered group. **F** Correlations of RNF43 alternations with PFS in TCGA pan-cancer cohort. **G** Correlations of RNF43 alternations with DSS in TCGA pan-cancer cohort. **H** Correlations of RNF43 alternations with OS in TCGA pan-cancer cohort. **I** Correlations of RNF43 alternations with mutation count in TCGA pan-cancer cohort. **J** Correlations of RNF43 alternations with TMB in TMB and immunotherapy cohort. **K** Correlations of RNF43 alternations with mutation count in TMB and immunotherapy cohort. **L** Correlations of RNF43 alternations with TMB in TMB and immunotherapy cohort

We further illustrated the genetic mutation characteristics of RNF43 in pan-cancer in the cBioPortal online database. As illustrated in Fig. 3A, the average alteration frequency of RNF43 in pan-cancer was high as 4%. Truncating mutation, amplification, and missense mutation were the main types of frequent genetic alterations of RNF43 in pan-cancer. In addition, our results showed that patients with endometrial carcinoma, esophagogastric adenocarcinoma, and colorectal adenocarcinoma had the top highest frequency of RNF43 alterations (Fig. 3B). In addition, the putative copy-number alterations of RNF43 from genomic identification of significant targets in cancer (GISTIC) included many types, such as gain function, diploid, and shallow deletion as displayed, which subsequently contributed to the changes of RNF43 expression (Fig. 3C). Furthermore, Fig. 3D showed the number, types, and sites of RNF43 gene modifications in pan-cancer. Between amino acids 0 and 783, G659V, P660S, and S661P were the most frequent mutation site, with 97 truncating mutations. Genetic mutations co-occurrence analysis revealed that the genetic alterations of TTN, AGAP10P, ALOX12P1, EIF2S2P4, SF3B6, MIR-7847/3P, OST4, AGBL5-AS1, GTF3C2-AS1, and SMAD5-AS1 were more common in the RNF43caltered group than those in the unaltered group (Fig. 3E). Besides, we discovered that cancer patients with RNF43-altered had a prolonged PFS time (p = 0.0133) (Fig. 3F) and DSS time (p = 0.0441) (Fig. 3G). To evaluate the potential effects of RNF43 on cancer immunotherapies, the TMB and Immunotherapy cohort in cBioPortal was then employed, and we detected that the RNF43-altered group had a longer OS time than the RNF43-unaltered group (p < 0.001), indicating that RNF43 genomic alternations might improve the immunotherapy efficacy in cancer treatment (Fig. 3H). In addition, the mutation count and TMB levels in the RNF43-altered group were significantly higher than those in the unaltered group in TCGA pan-cancer and TMB and Immunotherapy cohorts respectively (Fig. 3I–L).

### Prognostic significance of RNF43 in pan-cancer

**Fig. 4:**
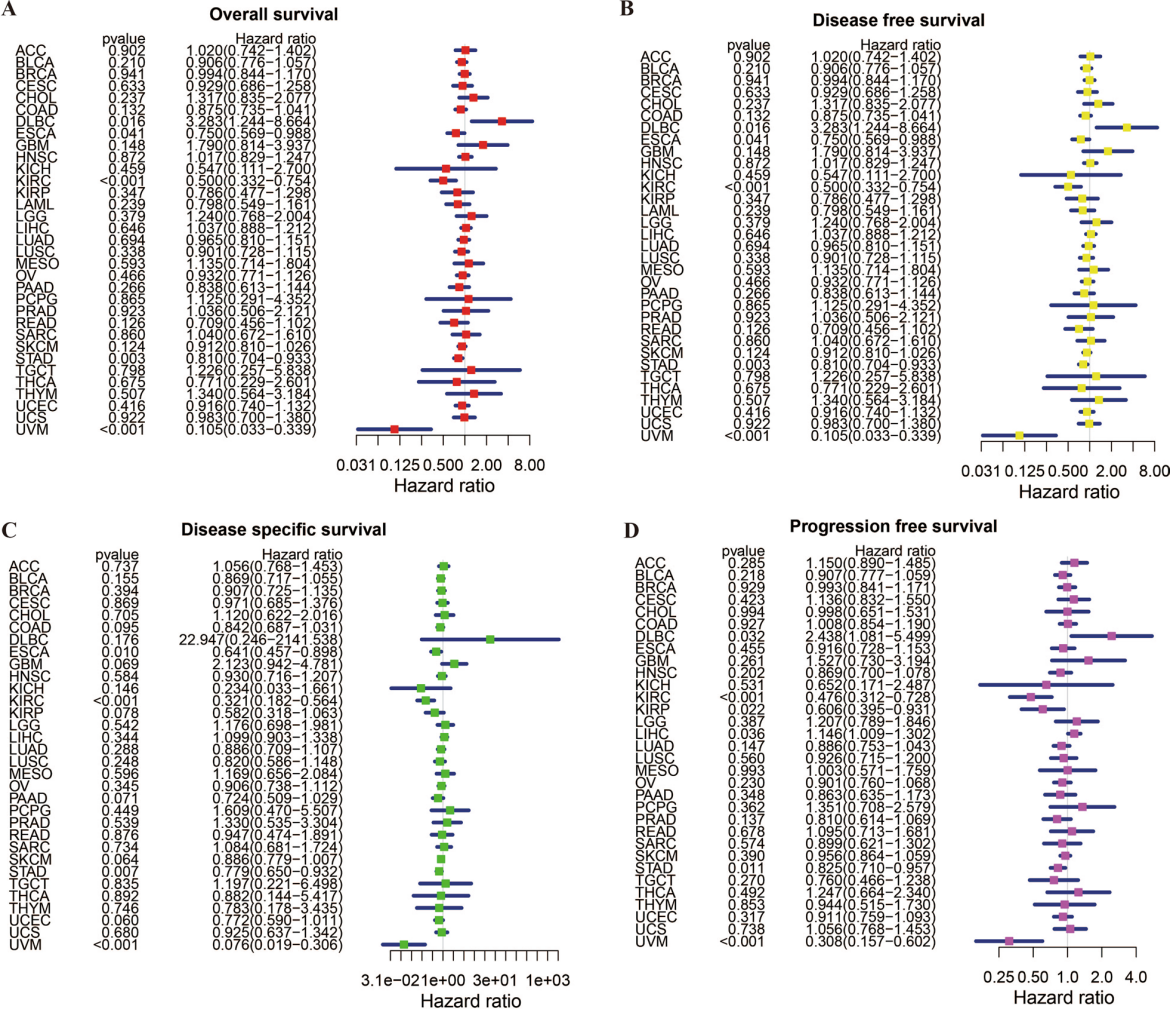
The forest plots of univariate Cox regression analyses of RNF43 in pan-cancer. The forest plot of (**A**) OS, (**B**) DFS, (**C**) DSS, and (**D**) PFS

To assess the predictive and prognostic value of RNF43 in pan-cancer, we conducted Cox regression analysis and Kaplan–Meier survival analysis to evaluate the relationship between RNF43 expression and cancer patients’ survival outcomes, including OS, DFS, DSS, and PFS. The Cox regression analysis results showed that RNF43 expression levels were related to OS in 5 tumors, related to DFS in 5 tumors, related DSS in 4 tumors, and related to PFS in 6 tumors (Fig. 4A–D). The results of univariate Cox regression analysis showed that the RNF43 expression levels were significantly related to OS in DLBC [(hazard ratio (HR), 3.283; 95% confidence interval CI 1.244–8.664; p = 0.016), ESCA (HR, 0.750; CI 0.569–0.988; p = 0.041), KIRC (HR, 0.500; CI 0.332–0.754; p < 0.001), STAD (HR, 0.810; CI 0.704–0.933; p = 0.003) and UVM (HR, 0.105; CI 0.033–0.339; p < 0.001)] (Fig. 4A). In these results, we indicated that RNF43 was a risk factor for DLBC patients, while it was a protective factor in ESCA, KIRC, STAD, and UVM. The DFS analysis demonstrated that RNF43 expression levels were strongly associated with DLBC [(HR, 3.283; CI 1.244–6.664; p = 0.016), ESCA (HR, 0.750; CI 0.569–0.988, P = 0.041), KIRC (HR, 0.500; CI 0.332–0.764; p < 0.001), STAD (HR, 0.810; CI 0.704–0.933; p = 0.003) and UVM (HR, 0.105; CI 0.033–0.339; p < 0.001)] (Fig. 4B), suggesting that RNF43 acted as a protective factor in ESCA, KIRC, STAD and UVM, while a risk factor in DLBC. The DSS analysis revealed that RNF43 plays an important role in patients with ESCA [(HR, 0.641; CI 0.457–0.898; p = 0.010), KIRC (HR, 0.321; CI 0.182–0.564; p < 0.001), STAD (HR, 0.779; CI 0.650–0.932; p = 0.007) and UVM (HR, 0.076; CI 0.019–0.306; p < 0.001)] (Fig. 4C). These findings indicated that RNF43 serves as a protective factor in ESCA, KIRC, STAD, and UVM. In addition, the PFS analysis indicated that RNF43 performed as a risk role in patients with DLBC [(HR, 2.438; CI 1.081–5.499; p = 0.032) and LIHC (HR, 1.146; CI 1.009–1.302; p = 0.036), and a protective role in patients with KIRC (HR, 0.476; CI 0.312–0.728; p < 0.001), KIRP (HR, 0.606; CI 0.395–0.931; p = 0.022), STAD (HR, 0.825; CI 0.710–0.957; p = 0.011) and UVM (HR, 0.308; CI 0.157–0.602; p < 0.001)] (Fig. 4D).

**Fig. 5:**
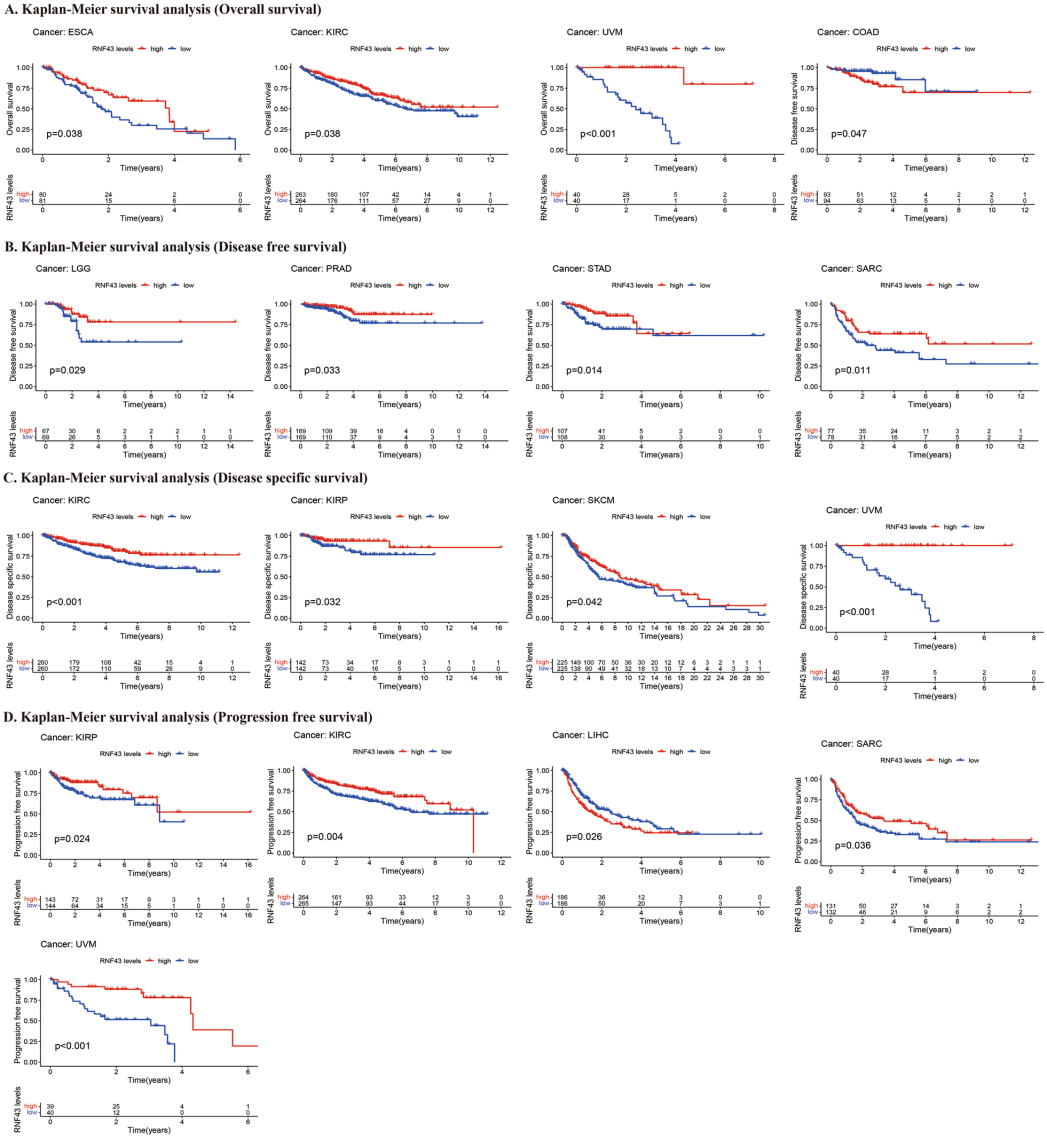
Kaplan–Meier survival curves comparing differential survival time between the high and low RNF43 expression groups in pan-cancer. The Kaplan–Meier survival curves of (**A**) OS, (**B**) DFS, (**C**) DSS, and (**D**) PFS

Furthermore, Kaplan–Meier cumulative curves showed that RNF43 expression levels were related to OS in 3 cancers, DFS in 5 cancers, DSS in 4 cancers, and PFS in 5 cancers (Fig. 5). Our results of Kaplan–Meier OS analysis showed that among patients ESCA (p = 0.038), KIRC (p = 0.038), and UVM (p < 0.001), high expression levels of RNF43 were correlated with good OS (Fig. 5A). The DFS analysis demonstrated that in patients with brain lower grade glioma (LGG) (p = 0.029), PRAD (p = 0.033), STAD (p = 0.014), and SARC (p = 0.011), high RNF43 expression levels were associated with a longer DFS time. However, in patients with COAD, high RNF43 expression levels were correlated with poor DFS (p = 0.047) (Fig. 5B). The analysis of DSS revealed the associations between high RNF43 expression levels and good prognosis in patients with KIRC (p < 0.001), KIRP (p = 0.032), SKCM (p = 0.042), and UVM (p < 0.001) (Figs. 5C). Besides, the PFS analysis indicated that patients with KIRP (p = 0.024), KIRC (P = 0.004), SARC (p = 0.036), and UVM (p < 0.001) of low RNF43 expression levels had a shorter survival time, which was opposite in patients with LIHC (p = 0.026) (Figs. 5D). Overall, our findings suggested that RNF43 was associated with OS in UVM, KIRC, and ESCA, associated with DFS in STAD, associated with DSS in KIRC and UVM, associated with PFS in KIRC, UVM, KIRP, and LIHC, confirming the potential role of RNF43 as a promising prognostic biomarker in human cancers.

### GSEA of RNF43 in pan-cancer

**Fig. 6:**
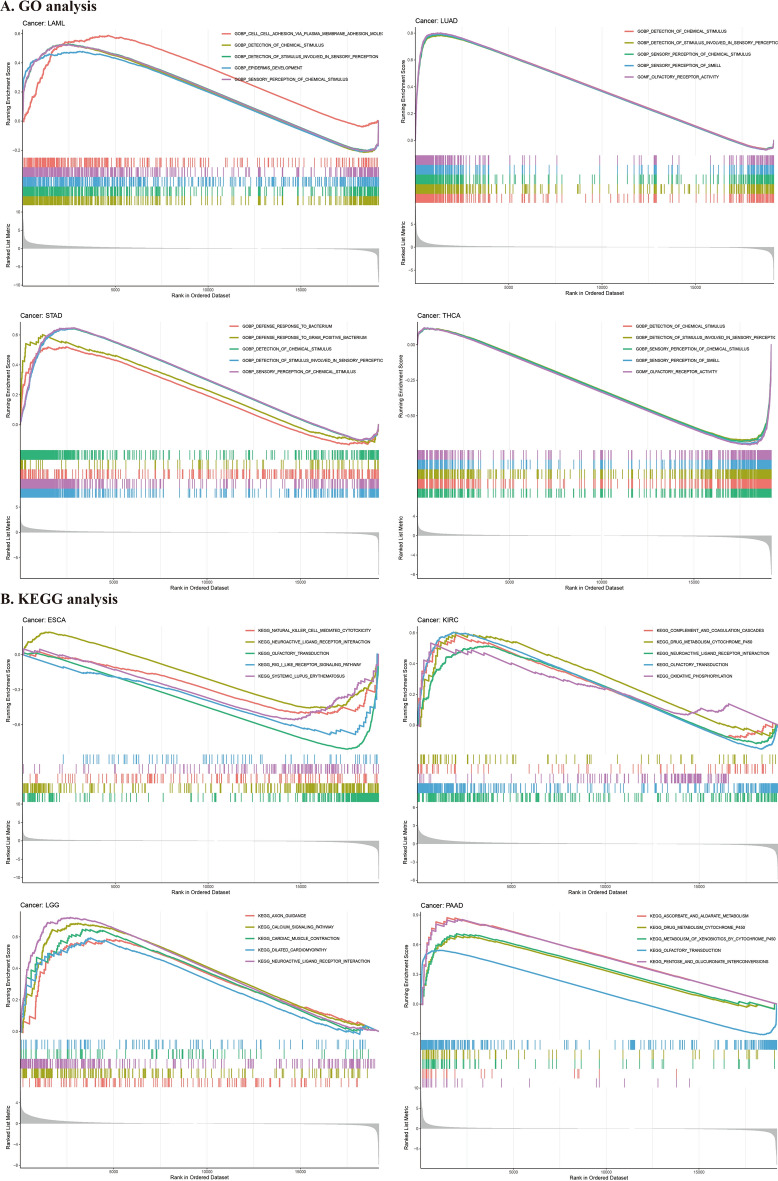
GSEA of RNF43 in pan-cancer. **A**–**D** GO analysis of RNF43. **E**–**H** KEGG pathway analysis of RNF43. Curves of different colors show different functions or pathways regulated in different cancers. Peaks on the upward curve indicate positive regulation and peaks on the downward curve indicate negative regulation

To deeply uncover the potential biological functions and signaling pathways that RNF43 involves in cancer progression, GESA, including GO functional annotation (Fig. 6A–D, Additional file 1: Figure S1) and KEGG pathway analysis (Fig. 6E–H, Additional file 1: Figure S2) was further performed. The results suggested that RNF43 might exert various biological functions, including the detection of chemical stimulus, detection of stimulus involved in sensory perception, and sensory perception of chemical stimulus in diverse cancer types, such as adrenocortical cancer (ACC) and LUAD. Also, RNF43 may participate in the regulation of immune response, immune system function, and immune cell infiltration during cancer development. In addition, KEGG analysis revealed that olfactory transduction was the most common signaling pathway of RNF43 participating in multiple cancer types. Besides, drug metabolism cytochrome P450 and neuroactive ligand-receptor interaction were also critical signaling pathways modulated by RNF43 in pan-cancer. These results indicated that RNF43 might be critically involved in regulating the tumor immune microenvironment, drug metabolism, and signal transduction in human cancers.

### Correlation of RNF43 with the tumor immune microenvironment in pan-cancer

**Fig. 7:**
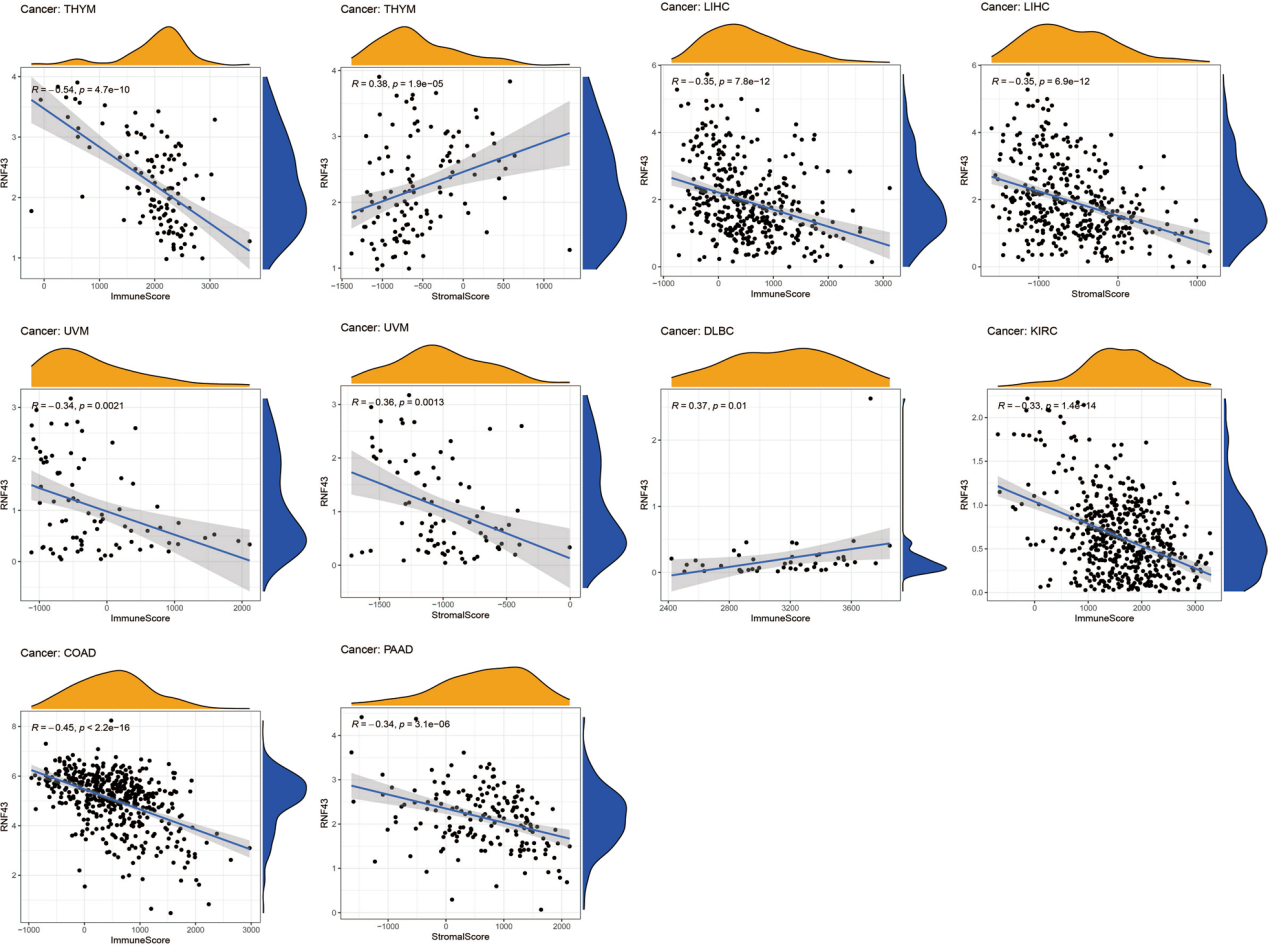
Correlation of RNF43 expression with immune score in 6 cancer types and stromal score in 4 cancer types by ESTIMATE algorithm

**Fig. 8:**
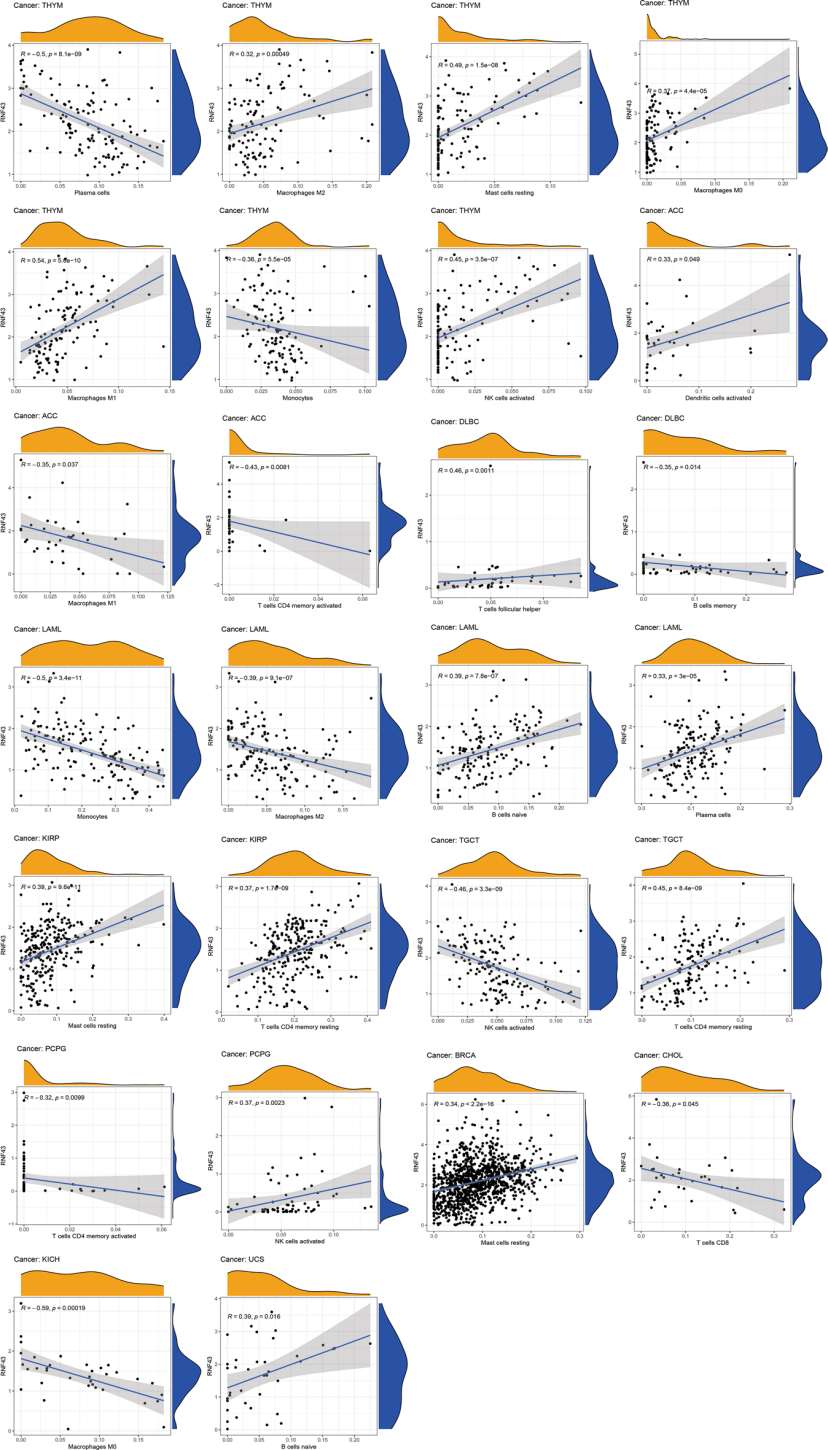
Correlations of RNF43 expression with the tumor immune microenvironment in TCGA pan-cancer. Analysis of immune-associated cells infiltration with RNF43 expression in pan-cancer

Next, we explored whether RNF43 could predict immune phenotypes in human cancers. First, we utilized the ESTIMATE to evaluate the correlations of RNF43 with immune and stromal scores (Fig. 7). The results suggested that RNF43 was negatively correlated with the immune score in COAD (p < 2.2e-16), KIRC (p = 1.4e-14), LIHC (p < 7.8e-12), thymoma (THYM) (p = 4.7e-10) and UVM (p = 0.0021), but was positively correlated with the immune score in DLBC (p = 0.01). Meanwhile, the results displayed a negative correlation between RNF43 expression and stromal score in LIHC (p = 6.9e–12), PAAD (p = 3.1e–06), and UVM (p = 0.0013), whereas a positive association between RNF43 expression and stromal score in THYM (p = 1.9e–05). We further studied the correlations between RNF43 expression and infiltrating immune cells. It was shown that RNF43 expression was significantly associated with various types of infiltrating immune-associated cells including dendritic cells, CD4+ T cells, CD8+ T cells, plasma cells, macrophages, B cells, and so on in diverse cancers (Fig. 8). Particularly, our results disclosed that the expression level of RNF43 was correlated with 7 types of immune-associated cells in THYM, including plasma cells, macrophages, mast cells resting, macrophages M0, macrophages M1, monocytes, and NK cells activated. Besides, the results also demonstrated that the expression of RNF43 was distinctly correlated with the immune-associated cell infiltration levels of macrophages in 4 types of cancer. The result revealed that the RNF43 expression was positively associated with the abundance of macrophages M2, macrophages M0, and macrophages M1 in THYM, while it was negatively associated with the abundance of macrophages M1 in ACC, macrophages M2 in LAML, and macrophages M0 in KICH. Moreover, we also detected that the expression of RNF43 was positively correlated with the infiltration level of mast cells resting in THYM, KIRP, and BRCA, NK cell activated in THYM and PCPG, dendritic cells activated in ACC, T cells follicular helper in DLBC, B cells naive in LAML and UCS, plasma cells in LAML and T cells CD4 memory resting in KIRP and TGCT, while it was negatively correlated with the abundance of plasma cells in THYM, monocytes in THYM and LAML, T cells CD4 activated in ACC and PCPG, B cells memory in DLBC, NK cells activated in TGCT and T cells CD8 in CHOL.

**Fig. 9:**
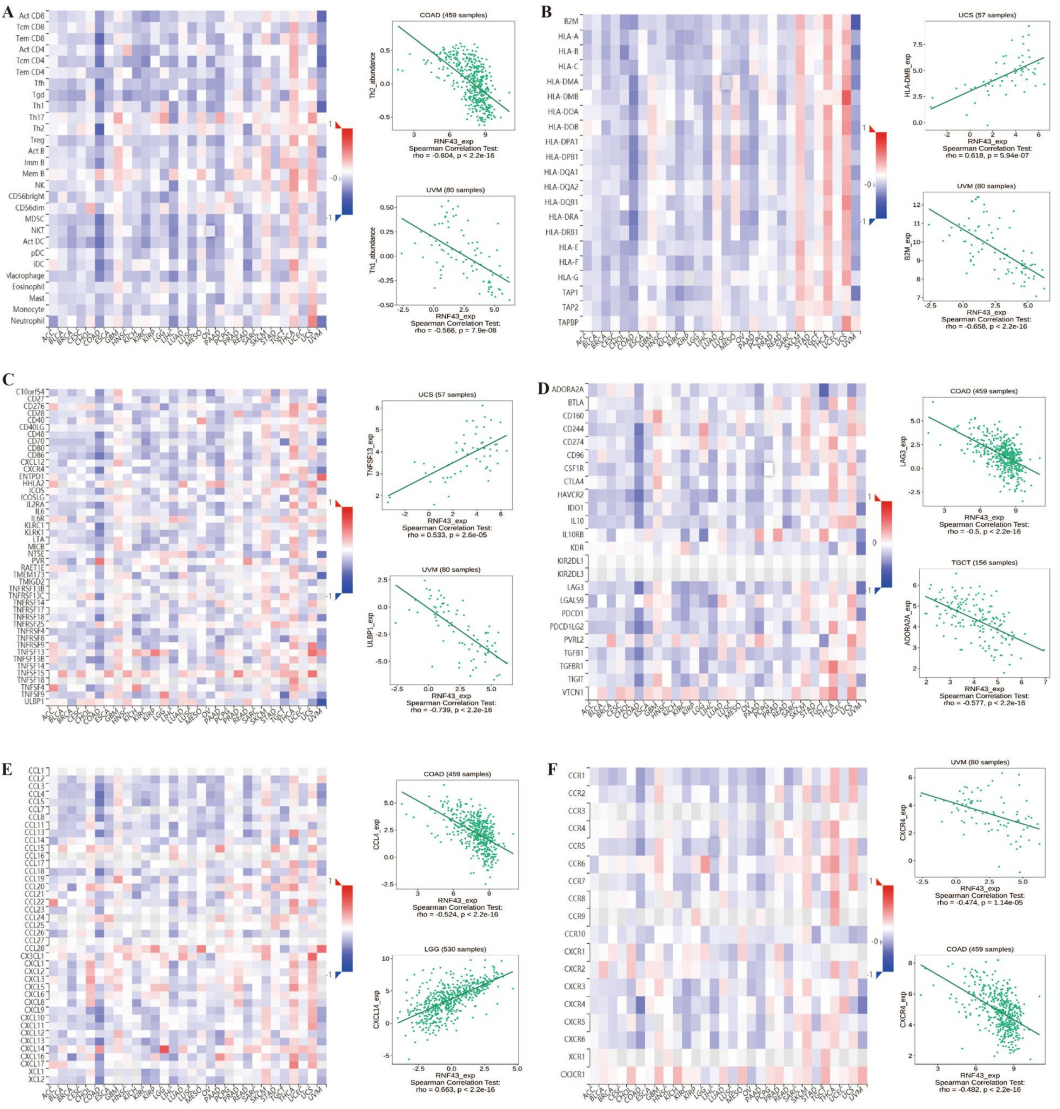
Correlations of RNF43 expression with the expression of immunomodulators in the TISIDB database. **A**–**F** Correlations between the expression of RNF43 and (**A**) TIL, **B** MHC molecules, **C** immune stimulator, **D** immune inhibitor, **E** chemokine, and (**F**) chemokine receptor. Red and blue represent positive and negative correlations, respectively

Furthermore, we explored the relationship between the expression of RNF43 and TIL, chemokine, chemokine receptor, major histocompatibility complex (MHC) molecules, immune stimulator, and immune inhibitor by gene co-expression analyses in the TISIDB database (Fig. 9). Our study demonstrated that RNF43 expression was significantly correlated with multiple TIL, such as Th1, Th2 and Tem CD8, MHC molecules, including HLA-DMB and B2M, immune stimulators such as CXCR4, PVR, TNFSF13 and ULBP1, immune inhibitors such as ADORA2A, CD244, LAG3 and TGFBR1, chemokines, such as CXCL14, CX3CL1, CCL28 and CCL4, and chemokines receptors including CXCR4 and CCR6 in multiple cancers. The analysis of TIL demonstrated that RNF43 expression was negatively related to Th1 in UVM and Th2 in COAD (Fig. 9A). Figure 9B displayed that the expression of RNF43 was negatively correlated with major histocompatibility complex B2M in UVM, while positively associated with HLA-DMB in UCS. As one of the 45 immune stimulators, the expression of RNF43 was positively correlated with TNFSF13 in USC (Fig. 9C). Meanwhile, in the analysis of 24 types of immune inhibitors, we observed a significant positive association between the expression of RNF43 and ADORA2A in TGCT (Fig. 9D). As illustrated in Fig. 9E, we have also studied 42 types of chemokines. The results showed a statistically positive correlation between the expression of RNF43 and CXCL14 in LGG and a statistically negative correlation between the expression of RNF43 and CCL4 in COAD. In the investigation of 18 types of chemokine receptors, we found that the expression of RNF43 was negatively correlated with CXCR4 in UVM and COAD (Fig. 9F). These findings indicated RNF43 has promising potential in predicting the immune-related phenotypes in different cancers.

### Correlation of RNF43 with MSI and TMB in pan-cancer

**Fig. 10:**
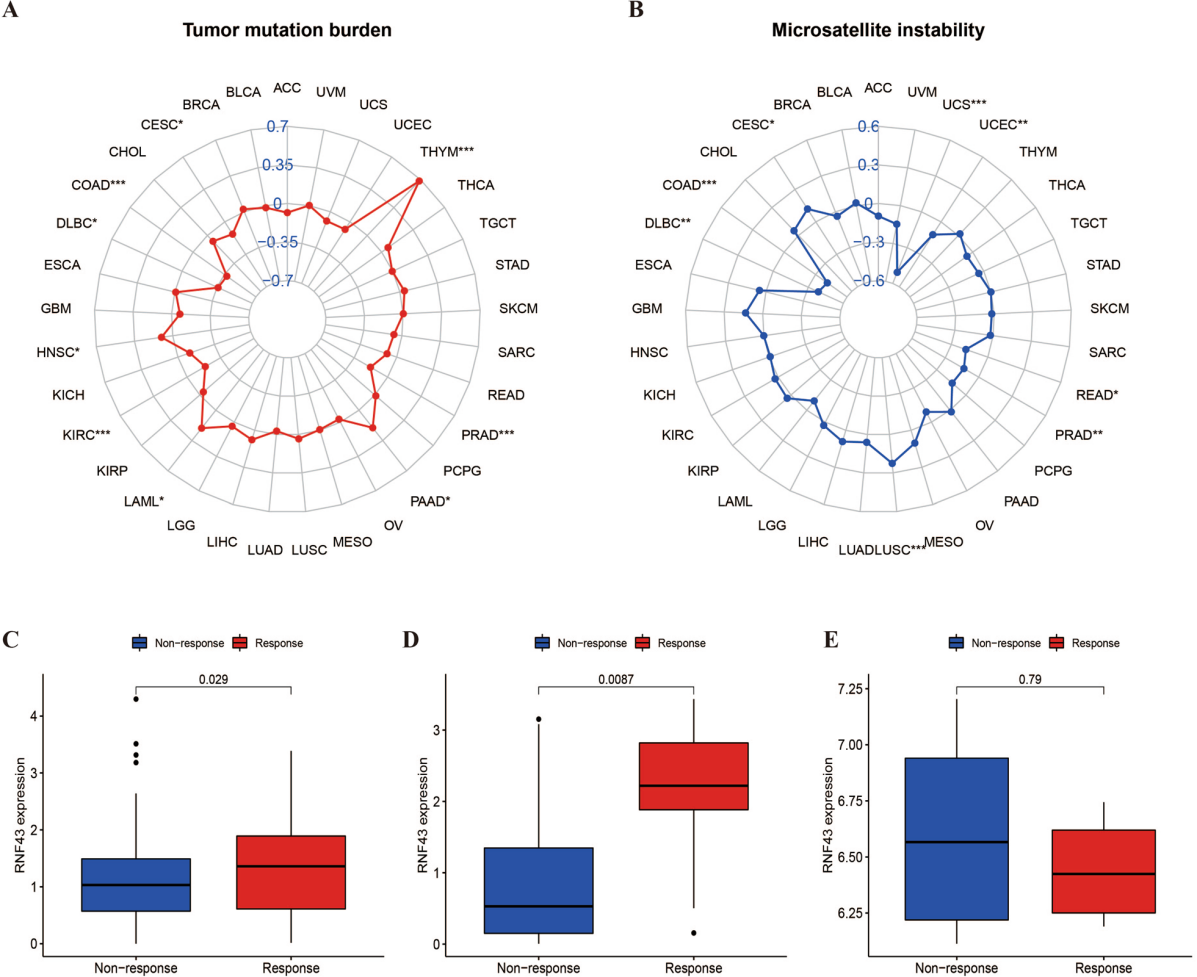
Correlations of RNF43 expression with TMB, MSI, and the immunotherapy efficacy. **A**, **B** Correlations between RNF43 expression and tumor mutation burden (TMB) and microsatellite instability (MSI) in pan-cancer. **C**–**E** Relationship between RNF43 expression and response to immunotherapy in three cohorts, **C** IMvigor, **D** GSE67501, and **E** GSE78220 *p < 0.05, p < 0.01, ***p < 0.001

Previous studies have shown that TMB and MSI levels are critical biomarkers for the immunotherapy efficacy in cancer management [18]. Therefore, we further investigated the relation of RNF43 expression with MSI and TMB in different cancers. The results showed that the correlation between RNF43 and MSI was remarkable in 8 cancer types. In particular, a statistically positive association between RNF43 expression and MSI was observed in CESC and LUSC, and a statistically negative association was detected in COAD, DLBC, PRAD, READ, UCEC, and UCS (Fig. 10A). With regards to TMB, it was demonstrated that RNF43 expression was positively correlated with TMB levels in HNSC, LAML, PAAD, and THYM, but was negatively associated with TMB levels in CESC, COAD, DLBC, KIRC, and PRAD (Fig. 10B). These findings suggested that RNF43 might be a potential biomarker in predicting the immunotherapeutic efficacy for cancer patients.

### Correlation of RNF43 with immunotherapeutic efficacy

To further verify the predictive value of RNF43 for immunotherapeutic efficacy, we extracted three datasets enrolling cancer samples that achieved immunotherapies from the GEO database. Our results found that patients with higher expression levels of RNF43 were more sensitive to anti-PD-1/PD-L1 treatment in the IMvigor210 cohort (p = 0.029) (Fig. 10C) and GSE78220 cohort (p = 0.0087) (Fig. 10D). On the contrary, no significant difference was found in the GSE67501cohort (p = 0.79) (Fig. 10E). These findings suggested that RNF43 could function as a promising predictor for anti-PD-1/PD-L1 treatment efficacy in clinical cancer management.

### Correlation analysis between RNF43 gene expression and drug sensitivity

**Fig. 11:**
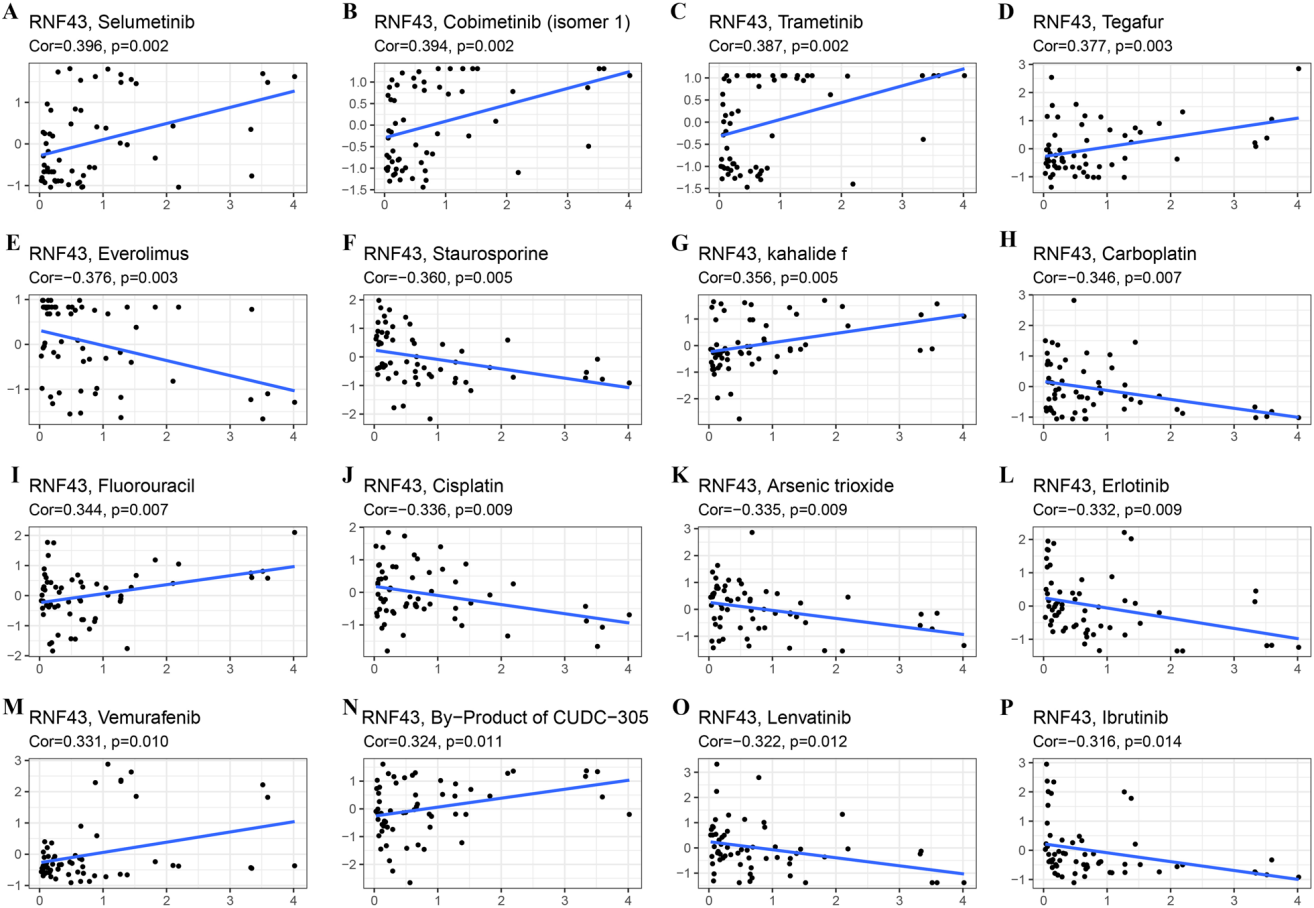
The correlation analysis between RNF43 and drug sensitivity in CellMiner. The top 16 anti-cancer drugs with statistical significance were displayed

Then, we also investigated the connection between RNF43 expression and drug sensitivity using the CellMiner^™^ database. In Fig. 11, we showed the most significant correlation of RNF43 with 16 top drugs. RNF43 expression was positively correlated with drug sensitivity of selumetinib (R = 0.396, p = 0.002), cobimetinib (R = 0.394, p = 0.002), trametinib (R = 0.387, p = 0.002), tegafur (R = 0.377, p = 0.003), kahalide f (R = 0.356, p = 0.005), fluorouracil (R = 0.344, p = 0.007), vemurafenib (R = 0.331, p = 0.010) and By-Product of CUDC-305 (R = 0.324, p = 0.011) (Fig. 11A-D, G, I, M, N). By contrast, RNF43 expression was negatively connected with drug sensitivity of everolimus (R = − 0.376, p = 0.003), staurosporine (R = − 0.360, p = 0.005), carboplatin (R = − 0.346, p = 0.007), cisplatin (R = − 0.336, p = 0.009), arsenic trioxide (R = − 0.335, p = 0.009), erlotinib (R = − 0.332, p = 0.009), lenvatinib (R = − 0.322, p = 0.012), and ibrutinib (R = − 0.316, p = 0.014) (Fig. 11E, F, H, J–L, O, P). The results confirmed that RNF43 expression was significantly correlated with the sensitivity of several anti-cancer agents, such as cisplatin, fluorouracil, and lenvatinib, suggesting the potential of RNF43 in predicting the chemotherapy and targeted therapy responses in cancer patients.

### IHC validation of RNF43

**Fig. 12:**
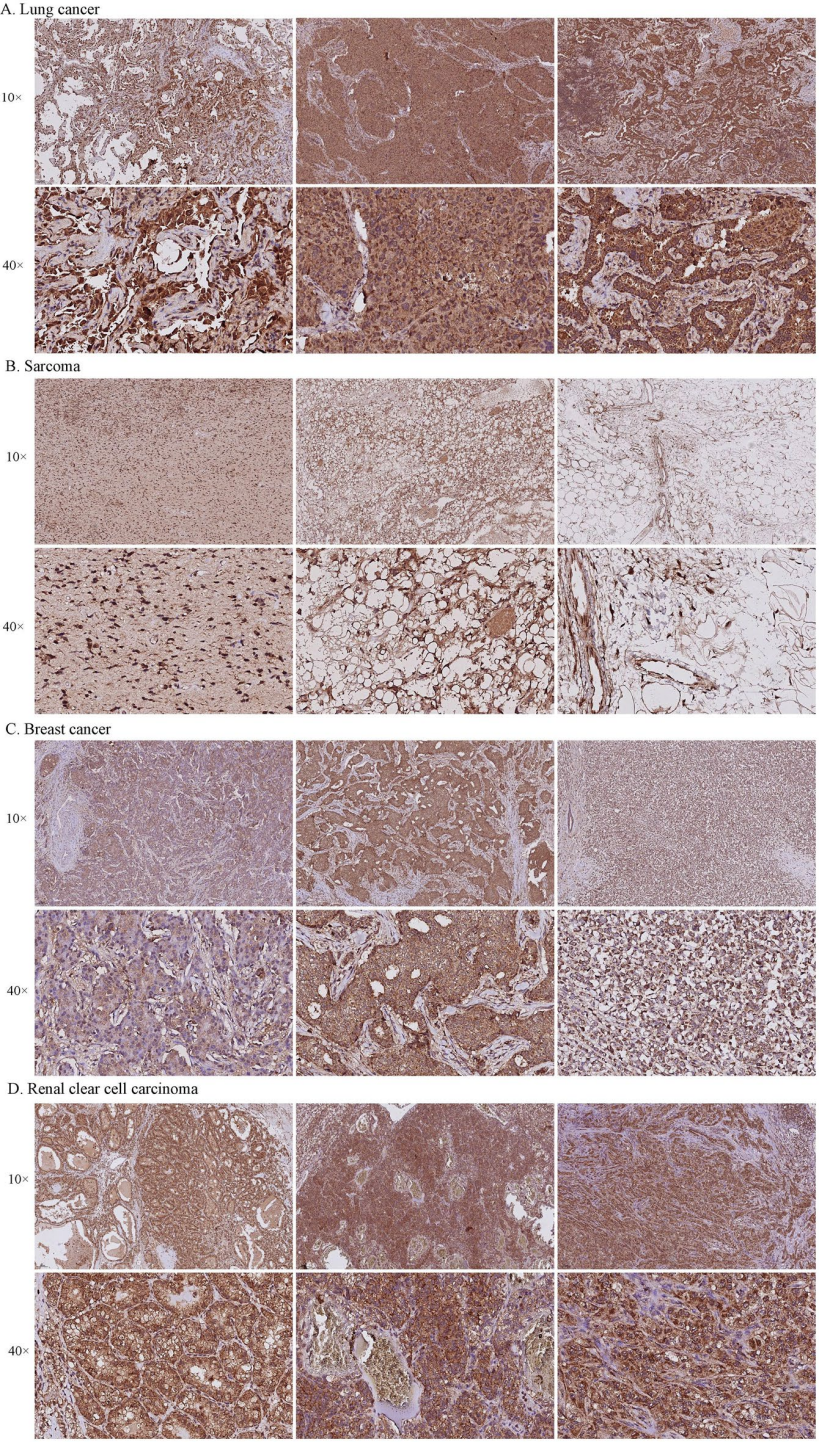
Immunohistochemistry images of RNF43 expression in tumor tissues. **A** Lung cancer, **B** Sarcoma, **C** Breast cancer, **D** Renal clear cell carcinoma

The expression of RNF43 was further validated by IHC among 4 different types of cancer by our cohorts, including breast cancer, lung cancer, kidney renal clear cell carcinoma, and sarcoma. As shown in Fig. 12, RNF43 was positively detected in all of the examined tumor tissue samples. A strongly positive expression of RNF43 was observed in kidney renal clear cell carcinoma, lung cancer, and breast cancer. Weakly positive RNF43 expression was detected in sarcoma patients. These findings were consistent with previous findings, and further validated the expression levels of RNF43 in human cancers.

## Discussion

In recent years, pan-cancer analysis has been of great concern because it can reflect the panorama of human cancers better [19]. Previous studies have proved that RNF43 expression was commonly downregulated in cancer patients, and RNF43 can serve as a critical tumor suppressor that inhibits various malignant behaviors of cancer cells, such as proliferation, invasion, stemness, epithelial-mesenchymal transition, and drug resistance [20]. Besides, RNF43 is frequently mutated in colorectal, endometrial cancers, and pancreatic cancer, which can deeply affect the behavior of cancer cells and induce cancerogenesis and progression [21, 22]. These results indicated that RNF43 plays important roles in tumorigenesis and development, which exhibit promising clinical significance and potential as the novel therapeutic target in the treatment of human cancers. However, a comprehensive investigation of RNF43 is lacking, and the functions of RNF43 in modulating the tumor immune microenvironment and predicting the immunotherapeutic efficacy need to be answered. Hence, our first and comprehensive pan-cancer research focused on the potential roles of RNF43 in predicting the prognosis, tumor immune phenotypes, and the response to immunotherapies and drug sensitivity.

We first investigated the expression-based panoramic picture of RNF43 in different cancer types. We found that the expression of RNF43 was significantly upregulated in several cancer types, such as COAD, LUAD, and STAD, while was significantly downregulated in some cancer types, including GBM, KIRP, and PCPG. These findings are consistent with the existing studies that have reported that RNF43 was upregulated in LUAD [23] and COAD [24]. Previous studies have found that the overexpression of RNF43 can directly ubiquitinate E-cadherin, leading to its degradation and thus potentiating metastasis of lung adenocarcinoma through inducible epithelial-to-mesenchymal transition (EMT) [23]. By single-cell RNA sequencing (scRNA-seq) analysis of primary gastric adenocarcinoma, Zhang et al. have identified 5 cell populations with distinct expression profiles and identified that RNF43 was expressed in a type of well-differentiated gastric adenocarcinoma, GA-FG-CCP which has been reported to be stained positive for PGA3 and MUC6, with the activation of the Wnt/β-catenin signaling pathway in previous studies [25]. Meanwhile, RNF43 expression was higher in earlier clinical-stage patients than that in an advanced clinical stage in KIRC, KIRP, and UVM. These findings indicated that RNF43 may help the early diagnosis of cancer patients. Furthermore, through Cox regression analysis and curves, we concluded that the high RNF43 expression predicted better survival outcomes in KIRC, UVM, STAD, ESCA, and KIRP, whereas the high expression of RNF43 indicated poor prognosis in DLBC and LIHC. Generally, the expression pattern and prognostic value of RNF43 were distinguished among different cancer types, and RNF43 can function as a critical prognosis-related factor in several cancers.

Several previous studies have identified RNF43 mutations in diverse cancers, such as colorectal cancers [21], pancreatic ductal adenocarcinoma [26], gastric cancer [27], and intrahepatic cholangiocarcinoma [28]. Previous studies have confirmed the high frequency of RNF43 mutations in colorectal cancer patients, which may play an activating role in the Wnt pathway in colorectal cancer [21]. The mutations of RNF43 eventually enhance colorectal cancer tumor growth and promote a high recurrence rate in patients [29]. In pancreatic cancer cell lines with RNF43 loss-of-function mutation, the frizzled level was no longer inhibited. Therefore, the Wnt/β-catenin signaling in acinar cells was increased, which may provide an additional stimulus to facilitate tumorigenesis and development [22]. The loss-of-function mutations of RNF43 also dampen the activation of DNA damage response, thus preventing apoptosis in gastric cells, and eventually leading to gastric carcinogenesis [30]. A recent study has presented that RNF43/ZNRF3 depletion mutation can contribute to live cancer tumorigenesis by modulating the differentiation of hepatocytes and the liver lipid metabolic state [10]. Prostate cancer is the most common non-cutaneous cancer in men worldwide, and multiple studies have detected RNF43 mutations in prostate cancer [31, 32]. Copy number loss of 17q22 is correlated with lower RNF43 and SRSF1 expression, enzalutamide resistance, and poor prognosis in prostate cancer [33]. By the investigation of RNF43 mutations on the cBioPortal database, we discovered that RNF43 possessed a high mutation rate of 4%, and truncating mutation was the most alternation type of RNF43 in pan-cancer. We also observed the co-occurrence of several genes, such as TTN, AGAP10P, ALOX12P1, and EIF2S2P4 was more frequent in the RNF43 alteration group. Most importantly, the alteration of RNF43 was significantly associated with PFS and DSS in cancer patients, and among patients who achieved immunotherapies, RNF43-altered patients had a longer OS time. The data unearthed the fact again that specific gene mutations may predict the prognosis and the responses to immunotherapy of cancer patients [34]. Our findings provide the characteristics of RNF43 mutations in pan-cancer for further understanding the roles of RNF43 mutations in cancer progression and treatment.

Immune cells are critical constituents of the tumor immune microenvironment, and have a remarkable influence on cancer development and survival [35, 36]. Immunotherapy is a new cancer treatment strategy through harnessing the immune cells to kill the cancer cells, and the plasticity of immune cell infiltration may contribute to the immunosuppressive microenvironment and immunotherapeutic resistance [37]. Therefore, investigating the correlation between biomolecules and immune cell infiltration can help predict the clinical outcomes and immunotherapy responses [38]. In this present study, our findings indicated that the expression of RNF43 was related to several immune cells, such as dendritic cells, T cells CD4, T cells CD8, plasma cells, macrophages, and B cells in various cancer types. RNF43 expression was significantly correlated with the immune-associated cell infiltration levels of macrophages in ACC, KICH, LAML, and THYM, as well as the infiltration levels of T cells CD4+ in ACC, KIRP, PCPG, and TGCT. CD4+ T cells and CD8+ cytotoxic T lymphocytes (CTLs) are critical immune cells that play critical roles in cancer development and immunity. Existing studies suggest that T cells CD4+ can target tumor cells in direct or indirect ways, either by eliminating tumor cells through the complex mechanisms, including the regulation of the tumor immune microenvironment, affecting antigen presentation, co-stimulation, and T cell homing [39, 40]. Our findings showed that RNF43 expression level was positively correlated with the infiltration of T cells CD4+ in KIRP, which is consistent with the mentioned result that a higher expression level of RNF43 is a protective factor in KIRP. In addition, tumor-associated macrophages are also critically involved in mediating tumor cells intrinsic properties and the tumor microenvironment remodeling, and macrophage polarization plays a prognostic role in multiple cancer types [41]. Therefore, our findings suggest that RNF43 expression may influence the cancer patients’ prognosis through the interaction with the infiltration of T cell CD4+ and T cell CD8+ and macrophage polarization during cancer progression.

Furthermore, we investigated the association between RNF43 expression and the expression levels of immune-related modulators. We detected that RNF43 expression was positively correlated with CXCL14 in LGG, which is mainly involved in regulating immune cell infiltration and serves as a potential mediator of the immune response [42]. A recent study has indicated that CXCL14 can recruit and activate CD8+ T cells in vitro and in vivo, and favor the anti-tumor CD8+ T-cell response in LGG [43]. It has been proved that Th1 and Th2 cells play a significant role in predicting the prognosis of cancer patients [44]. Our study found that RNF43 expression was negatively related to Th1 in UVM and Th2 in COAD, indicating the potential functions of RNF43 in regulating Th1/2 cells in these cancers. Besides, a positive correlation of RNF43 expression with TNFSF13 and ADORA2A was also observed in our investigation. Previous research has clarified that TNFSF13 is closely associated with cancer occurrence and development, and it may be an effective biomarker for predicting the immunotherapy response [45]. It has also been confirmed that ADORA2A acts as an intrinsic negative regulator in the procedure of NK cell maturation and the anti-tumor immune response [46]. Above all, RNF43 may interact with various immune-related biomolecules in different cancers, thereby regulating the tumor immune microenvironment and cancer progression.

The results of the GSEA analysis showed that RNF43 was obviously involved in detection of chemical stimulus, detection of stimulus involved in sensory perception, and sensory perception of chemical stimulus in pan-cancer. Besides, olfactory transduction, drug metabolism cytochrome P450, and neuroactive ligand receptor interaction were identified as the most three common signaling pathways in RNF43 biological function in pan-cancer analysis. Previous studies have confirmed that RNF43 is an E3 ubiquitin ligase, and is a critical negative feedback regulators of the Wnt pathway, which plays significant roles in mediating drug resistance [47, 48]. Notably, cytochrome P450 can influence drug response through metabolism, affecting the therapeutic efficacy and toxicity of drugs [49]. These results indicated that RNF43 may be critically involved in mediating cancer immunity, drug metabolism and stimulus sensory in diverse cancers.

Recently, immunotherapies have been widely accepted as an effective treatment approach for cancer patients [50]. However, many cancer patients have no or limited responses to immunotherapies and may gradually develop immunotherapeutic resistance during treatment. It has been observed that higher TMB level can work as an independent predictor of better responses to immunotherapy in multiple cancers [51, 52]. In addition, MSI can also serve as an effective positive predictor of cancer immunotherapy [53]. Previous research has indicated that RNF43 mutations are closely correlated with MSI, TMB, and mismatch repair deficiency (dMMR) phenotype in colon cancer patients, indicative of the promising potential of the combination of immune checkpoint inhibitors with Wnt/β-catenin signaling pathways inhibitors as a reasonable therapeutic strategy in cancer treatment [54]. Our study confirmed that RNF43 expression is significantly correlated with MSI and TMB in several cancer types, such as CESC, LUSC, COAD, and DLBC. These results display that RNF43 may have the potential as a promising biomarker associated with immunotherapy resistance and predict immunotherapeutic responses in cancer patients. To further confirm the effects of RNF43 on cancer immunotherapies, we analyzed the association between RNF43 expression and the immunotherapy efficacy in these datasets that consist of cancer patients who acquired immunotherapies. Our research revealed that cancer patients with high RNF43 expression had better responses to anti-PD-1/PD-L1 treatment. These findings further identify RNF43 as a critical biomarker for predicting the immunotherapeutic efficacy, and dynamic screening RNF43 expression may be an effective approach to predict the responses of cancer patients to immunotherapies.

Targeted therapies have been developed to inhibit specific molecules that are responsible for facilitating tumor growth. Understanding the molecular signatures and genetic mutations involved in cancer initiation and progression is fundamental for developing effective treatments for human malignancies [55, 56]. For example, Fibroblast Growth Factor Receptor 3 (FGFR3) mutations have been confirmed as potent oncogenic drivers in several cancers, and FGFR3 inhibitors may be a novel therapeutic strategy for patients with FGFR3-altered cancer [57]. Previous studies have indicated that RNF43 participates in mediating targeted therapy resistance and chemoresistance in several cancers, which may serve as a novel therapeutic target for overcoming drug resistance in cancer management [22]. In melanoma, RNF43 has been found to inhibit cell invasion and reverse the resistance to BRAF V600E and MEK inhibitors by stimulating the ubiquitination and proteasomal degradation of VANGL2 and inhibiting ROR2 in vitro and in vivo [58]. It has been found that RNF43 depletion can impair the sensitivity to γ-radiation and chemotherapy by suppressing the activation of DNA damage response via directly targeting phosphorylated H2A histone family member X (γH2AX) in gastric cancer [30]. In clear cell renal cell carcinoma, RNF43 can inhibit malignant behavior and reverse pazopanib resistance by inhibiting the YAP signaling by decreasing YAP phosphorylation via p-LATS1/2 [59]. By employing the CellMine^™^ database, we found that RNF43 expression was significantly correlated with the sensitivity of several drugs that have been widely applied in cancer clinical treatment or clinical trials, such as cisplatin, erlotinib, cobimetinib, and everolimus. These results further verify that RNF43 exhibits great potential as a predictive biomarker for the response to anti-cancer agents and a promising therapeutic target for overcoming drug resistance. Besides, dynamic monitoring of RNF43 expression may be an effective approach to evaluate the responses of cancer patients to anti-cancer drugs, thus helping develop personalized treatment strategies for individual cancer patients.

However, our study also has several limitations. Firstly, we only validated the expression pattern of RNF43 with our clinical samples, but the clinicopathological significance of RNF43 is assessed based on online databases, which needs to be further verified in our cohorts in the future. In addition, there is a lack of experimental validation for the predicted roles of RNF43 in mediating the tumor immune microenvironment. Besides, large-scale cohorts are urgently warranted to explore the predictive value of RNF43 in pan-cancer outcomes and immunotherapy efficacy, which is time-consuming.

## Conclusions

In conclusion, the pan-cancer analysis comprehensively reveals that RNF43 can effectively predict the prognosis, immune-related phenotypes, and immunotherapy efficacy in human cancers. Hence, RNF43 plays a key role in modulating the tumor immune microenvironment, and serves as a prognostic, therapeutic and immunological biomarker and a novel therapy target in clinical cancer management.

### Supplementary Information

**Additional file 1:** **Figure S1.** GO analysis of RNF43 in pan-cancer. RNF43 may regulate diverse biological functions in different cancers, such as the detection of chemical stimulus, detection of stimulus involved in sensory perceptic, and epidermis development. Curves of different colors show different functions regulated in different cancers. Peaks on the upward curve indicate positive regulation and peaks on the downward curve indicate negative regulation. **Figure S2.** KEGG pathway analysis of RNF43 in pan-cancer. RNF43 may participate in mediating various signaling pathways in different cancers, such as allograft rejection, antigen processing and presentation, and PPAR signaling pathway. Curves of different colors show different pathways regulated in different cancers. Peaks on the upward curve indicate positive regulation and peaks on the downward curve indicate negative regulation.

## Data Availability

All the original data and datasets presented in this study can be found in online repositories. The names of the repository/repositories and accession number(s) can be found in the article/Supplementary Material.
