# Supplementary material for: Pan-cancer analysis identifies RNF43 as a prognostic, therapeutic and immunological biomarker
Source: Eur J Med Res. 2023 Oct 17;28:438. doi: 10.1186/s40001-023-01383-1 (PMC10580550; doi:10.1186/s40001-023-01383-1)

Figure S1. GO analysis of RNF43 in pan-cancer. RNF43 may regulate diverse biological functions in different cancers, such as the detection of chemical stimulus, detection of stimulus involved in sensory perceptic, and epidermis development. Curves of different colors show different functions regulated in different cancers. Peaks on the upward curve indicate positive regulation and peaks on the downward curve indicate negative regulation.

Figure S2. KEGG pathway analysis of RNF43 in pan-cancer. RNF43 may participate in mediating various signaling pathways in different cancers, such as allograft rejection, antigen processing and presentation, and PPAR signaling pathway. Curves of different colors show different pathways regulated in different cancers. Peaks on the upward curve indicate positive regulation and peaks on the downward curve indicate negative regulation.


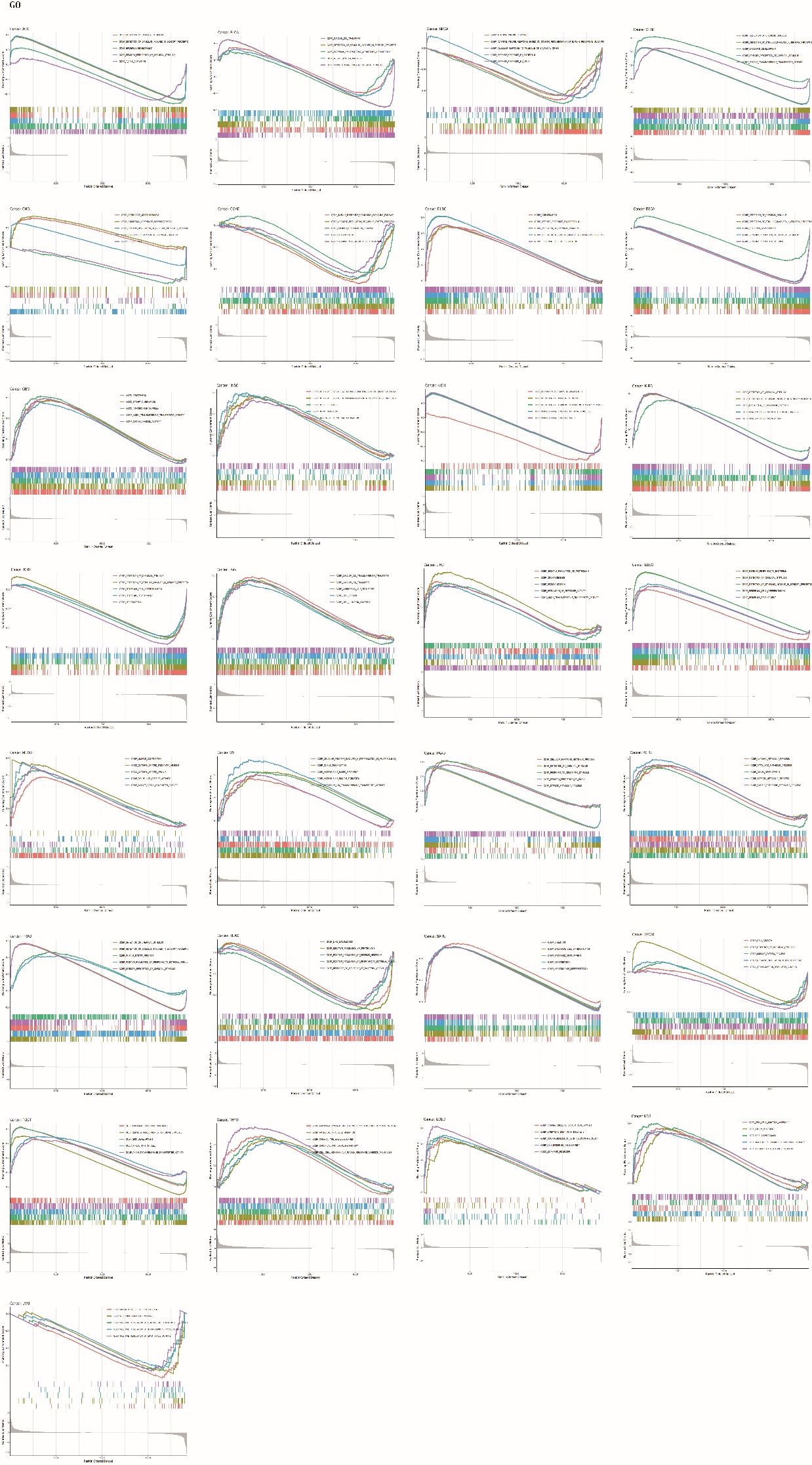


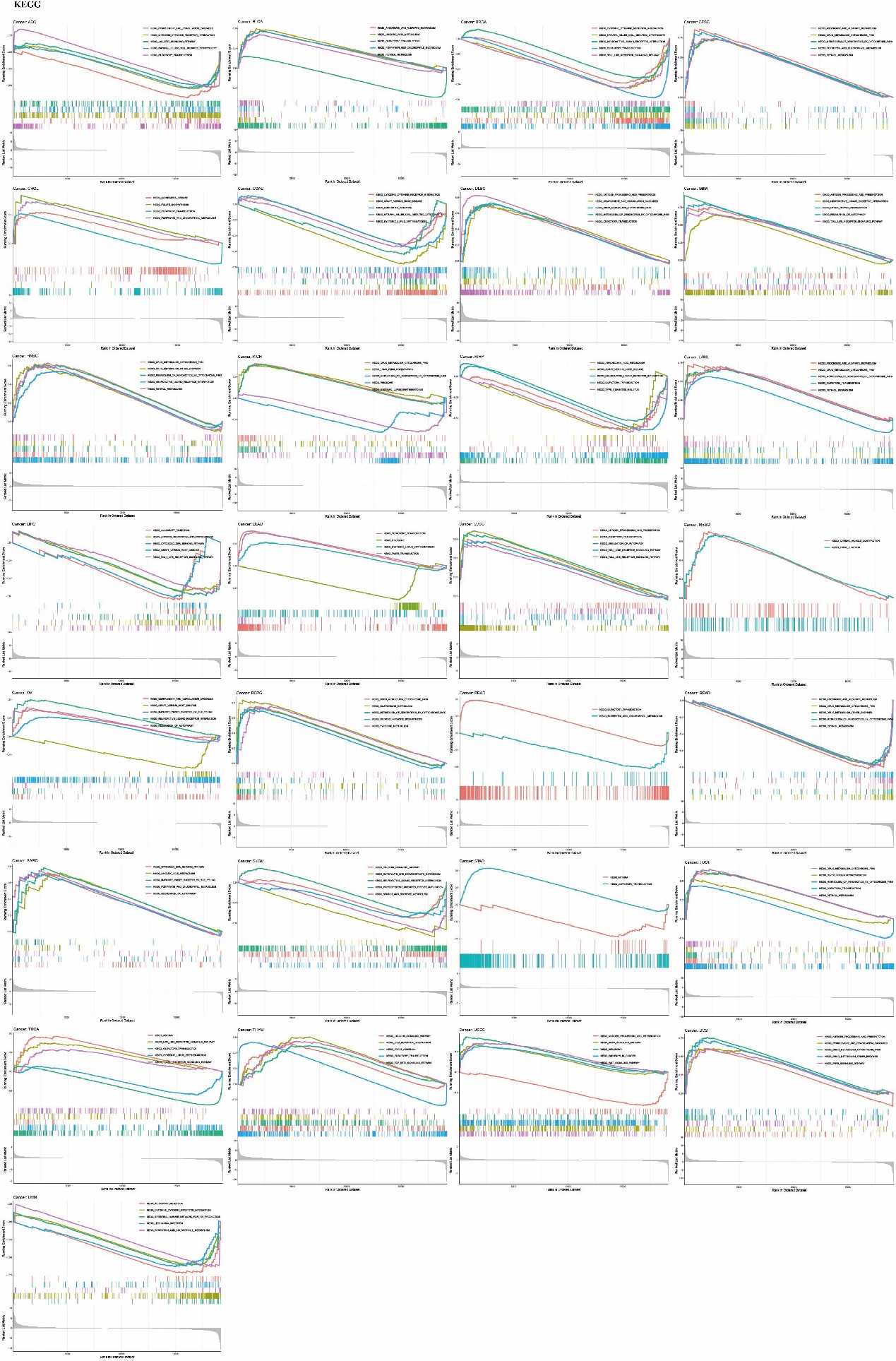

Supplement: Supplementary file 1 — Additional file 1: Figure S1. GO analysis of RNF43 in pan-cancer. RNF43 may regulate diverse biological functions in different cancers, such as the detection of chemical stimulus, detection of stimulus involved in sensory perceptic, and epidermis development. Curves of different colors show different functions regulated in different cancers. Peaks on the upward curve indicate positive regulation and peaks on the downward curve indicate negative regulation. Figure S2. KEGG pathway analysis of RNF43 in pan-cancer. RNF43 may participate in mediating various signaling pathways in different cancers, such as allograft rejection, antigen processing and presentation, and PPAR signaling pathway. Curves of different colors show different pathways regulated in different cancers. Peaks on the upward curve indicate positive regulation and peaks on the downward curve indicate negative regulation. [file 40001_2023_1383_MOESM1_ESM.docx]
